# Biogenic Gold Nanocrystals Knock Down *Pseudomonas aeruginosa* Virulence via Quorum-Sensing and Antibiofilm Potential

**DOI:** 10.3390/nano15211648

**Published:** 2025-10-28

**Authors:** Sanket Kumar, Balwant Singh Paliya, Brahma N. Singh, Shivankar Agrawal

**Affiliations:** 1 Pharmacology Division, CSIR—National Botanical Research Institute, Lucknow 226001, India; sanket.nbri@gmail.com (S.K.);; 2 APC Microbiome Ireland, School of Microbiology, University College Cork, T12 CY82 Cork, Ireland

**Keywords:** quorum sensing, anti-virulence, anti-bacterial, anti-biofilm, nanoparticles

## Abstract

Multidrug resistance has also been accompanied by the prolonged use of antibiotics that makes complications in treatment. Biofilm in pathogenic bacteria is the most serious challenge linked with chronic illnesses and also contributes to virulence and drug resistance. Several bacterial pathogens employ the Quorum-sensing (QS) mechanism to coordinate their collective behaviors like bioluminescence, virulence, and biofilm formation. Therefore, agents that inhibit or interfere with bacterial QS and biofilm formation are emerging as a new class of next-generation antibacterial. Recently, nanoparticles have been employed to improve the efficacy of existing antibacterial agents. In the present study, gold nanocrystals were synthesized by using *Koelreuteria paniculata* (KP) leaf extract. Synthesized nanocrystals were characterized by a face-centered cubic structure of ~20 nm by XRD, FTIR, Zeta sizer, and TEM. Biogenic Gold nanocrystals (BGNCs) exhibited extended QS inhibition in bio-indicator strains *Chromobacterium violaceum* and *Pseudomonas aeruginosa* biosensor strains. BGNCs strongly suppressed QS-controlled violacein production in *C. violaceum* CV026, and elastase, protease, pyocyanin, alginate, and biofilm formation in *P. aeruginosa* (PA01). In addition, BGNCs notably suppressed the relative expression of PA01 quorum sensing, biofilm-forming, and virulence-regulating genes, as quantified by qRT-PCR. As a result of the broad-spectrum suppression of QS and biofilm by BGNCs, it is anticipated that these nontoxic bioactive nanocrystals can be employed as surface sterilization agents in nosocomial infections.

## 1. Introduction

The increasing prevalence of multidrug resistance among pathogenic bacteria has become a major clinical concern, underscoring the urgent need for novel and more potent antimicrobial agents to combat drug-resistant infections [1]. Biofilm formation, one of the key virulence factors in bacterial pathogens, further contributes to antimicrobial resistance [2]. In Gram-negative bacteria, the quorum sensing (QS) pathway a crucial mechanism regulating virulence and biofilm development is mediated by signaling molecules such as *N*-acyl homoserine lactones (AHLs) [3]. Since the discovery of QS, extensive research has revealed its pivotal role in regulating various biological functions, particularly biofilm formation [4]. Consequently, disrupting the QS system represents a promising strategy to interfere with biofilms and other QS-regulated virulence traits. Anti-QS strategies are therefore considered attractive alternatives for developing novel therapeutic agents to combat bacterial infections [5]. A key advantage of targeting QS is the reduced likelihood of inducing bacterial resistance, as these approaches inhibit virulence and biofilm formation without directly affecting bacterial growth [6]. In recent years, nanoparticles (NPs) have attracted considerable attention due to their diverse biomedical applications. Among them, gold nanoparticles (AuNPs) have garnered significant scientific interest because of their remarkable physical, chemical, and biocompatible properties [7]. The synthesis and application of AuNPs represent a rapidly evolving field within nanotechnology, particularly for managing microbial infections. Recent studies have demonstrated that AuNPs can inhibit the production of virulence factors and biofilm formation in several pathogenic bacteria, highlighting their potential as anti-virulence agents.

Although various physical and chemical methods are available for nanoparticle synthesis, many of these processes involve toxic reagents, high energy requirements, and complex purification steps, posing environmental and safety challenges [8]. As a result, there is growing interest in developing sustainable, cost-effective, and eco-friendly synthetic approaches. Biological synthesis methods utilizing natural materials such as plants, fungi, and bacteria have emerged as a promising alternative, as they are generally biocompatible and environmentally benign [9]. In particular, plant-mediated synthesis has gained prominence in recent years due to its simplicity and efficiency, eliminating the need for complex intracellular synthesis, multiple purification steps, prolonged incubation, or continuous maintenance [10].

Recent studies also have highlighted the efficacy of green synthesis methods for producing gold nanoparticles (AuNPs) using various biomaterials, such as natural sponges, lysozyme crystals, and other biotemplates. These approaches offer eco-friendly alternatives to traditional chemical methods, utilizing natural reducing agents and templates to fabricate nanoparticles with controlled sizes and shapes. For instance, natural sponges have been employed as templates for synthesizing AuNPs, leveraging their porous structures to support nanoparticle formation. This method not only provides a sustainable route for nanoparticle production but also enhances the catalytic properties of the resulting AuNPs, making them effective in the reduction in pollutants like 4-nitrophenol [11]. Similarly, lysozyme crystals, a type of protein crystal, have also been utilized in the green synthesis of AuNPs. The crystalline structure of lysozyme facilitates the controlled growth of AuNPs, resulting in nanoparticles with uniform sizes. These lysozyme-stabilized AuNPs exhibit significant antibacterial activity, demonstrating their potential for biomedical applications [12]. Additionally, other biotemplates, such as bacterial cellulose nanofibers, have been explored for AuNP synthesis. These nanofibers provide a scaffold for AuNP formation, leading to composites that are effective in biosensing applications due to their high surface area and biocompatibility [13].

In the present study, gold nanoparticles were synthesized using an aqueous leaf extract of *Koelreuteria paniculata* (KP). The phytoconstituents of *K. paniculata* are well known for their diverse biological activities, including antioxidant, antimicrobial, and anti-inflammatory properties [14]. Moreover, AuNPs themselves have been reported to exhibit anti-QS activity [2]. Therefore, it was hypothesized that the bio-fabrication of AuNPs using *K. paniculata* extract would yield nanoparticles with enhanced efficacy against biofilm formation and QS-regulated virulence in pathogenic bacteria. The synthesized nanoparticles were comprehensively characterized, and their anti-virulence and anti-biofilm activities were evaluated against the Gram-negative pathogen *Pseudomonas aeruginosa* (PAO1). The impact of these biofabricated nanoparticles on biofilm architecture was further examined using microscopic and spectroscopic analyses.

## 2. Materials and Methods

### 2.1. Culture Media, Chemicals, and Bacterial Strain

The media for microorganisms, including Luria-Bertani Broth (LB), Luria-Bertani agar (LA), and Nutrient Broth (NB), were obtained from HiMedia, Mumbai, India. Chloroauric acid (HAuCl_4_) was obtained from Sigma Aldrich, St. Louis, MO, USA and all other chemicals met analytical grade standards. *Pseudomonas aeruginosa* (PA01) was routinely grown in sterile Nutrient Broth (NB) culture media, whereas *Chromobacterium violaceum* 12472 ATCC was cultivated in sterile Luria Bertani (LB) medium with agitation at 120 rpm in a laboratory shaker. Bacterial cultures were preserved at −80 °C in LB medium containing 40% (*v*/*v*) glycerol. For experimental purposes, *P. aeruginosa* was cultured at 37 °C, and *C. violaceum* at 30 °C. Microbial cultures’ optical density (OD) was adjusted to 0.4 at A600 nm using sterile media for the experiments.

### 2.2. Preparation of Biogenic Gold Nanocrystals

Gold nanocrystals were prepared using the modified precipitation method [15]. Initially, KP aqueous leaf extract was prepared by soaking 10 g of dried leaf powder in 100 mL of double-distilled water, followed by fine grinding, washing, and drying before extraction. The extract was obtained by mixing the leaf powder with DDW in a conical flask and stirring for 2 h. Subsequently, the mixture was heated in a water bath at 60 °C for 45 min. The resulting solution was centrifuged at 8000 rpm for 15 min using a Thermo Fisher, Waltham, MA, USA Scientific centrifuge and filtered through a 0.45 μm PVDF syringe filter (SmartPor, Seoul, Republic of Korea).

For Biogenic Gold nanocrystals preparation, a 10^2^ μM chloroauric acid (HAuCl_4_) solution in a 250 mL conical flask was mixed with 10 mL of KP leaf extract dropwise, stirring continuously at room temperature with a magnetic stirrer. The pH was adjusted to approximately 8 using 1 M NaOH under dark conditions. The formation of BGNCs was indicated by a color change. BGNCs were obtained after 24-h incubation, followed by the solution being centrifuged at 12,000 rpm for 10 min, washed thrice with deionized water (DW) and ethanol, and air-dried. Bare gold nanocrystals (GNCs), or Chemogenic gold nanocrystals CGNCs, were synthesized chemically using a documented method [16].

### 2.3. Characterization of Nanocrystals

NCs’ physicochemical parameters were analyzed using advanced microscopic and spectroscopic techniques. UV-Vis’s spectroscopy confirmed metallic nanocrystal formation; further, metal ion reduction was validated across a range of 380–700 nm wavelengths using a UV-vis spectrophotometer Evolution 201 (Thermo Fisher Scientific, Waltham, MA, USA).

Particle shape and size distribution were examined using a transmission electron microscope (JEOL JEM-1400, TEM, Tokyo, Japan), operated at 40–120 kV [17]. Ethanolic colloidal samples (1 mg/mL) of nanocrystals were deposited on carbon-coated copper grids and dried for TEM analysis after ultrasonic dispersion. Surface characteristics and composition were studied using a chromium-coated sample analyzed with a high-resolution Quanta 250 scanning electron microscope (SEM, FEI, Netherlands) at 30 kV. Elemental composition was determined via energy-dispersive X-ray (EDX) analysis using an EDX analyzer.

The Anton Paar Litesizer-500 DLS analyzer determined NCs’ size distribution, polydispersity index (PDI), and zeta potentials (mV). Crystalline phase analysis used an X-ray diffraction instrument (SmartlabSERigaku, Tokyo, Japan) recording diffraction intensities from 20–80° at 2θ angles. Fourier-transform infrared spectroscopy (FT-IR) analyzed NCs samples mixed with potassium bromide (KBr) using a Nicolet 6700 FT-IR instrument (Thermo Scientific), recording spectra from 400–4000 cm^−1^ with 4 nm resolution.

### 2.4. Minimum Inhibitory Concentration (MIC)

Minimal inhibitory concentrations (MICs) of nanocrystals (NCs) were determined following CLSI 2015 guidelines. Test bacteria *C. violaceum* 12472 and *P. aeruginosa* PA01 (1 × 10^5^ cells/mL) were exposed to serially diluted NCs (5–120 µg/mL) in a 96-well microtiter plate. Plates were incubated at 37 °C for 24 h with orbital agitation, and bacterial growth was monitored at A600 nm. MIC was defined as the NC concentration inhibiting visible bacterial growth. This assay was performed in triplicate to ensure the accuracy and reliability of the results.

### 2.5. In Vitro Screening of BGNCs for Anti-QS Activity

To study the quorum-sensing (QS) inhibition by BGNCs, we followed [18] methodology using a standard disk diffusion assay with *C. violaceum* ATCC 12472. This bacterium produces a violet pigment in response to AHL-based QS. Overnight cultures were mixed with 0.3% molten LB agar and poured onto LB agar plates. Various concentrations of NCs were applied to a sterile paper disk at the center of each plate. Plates were incubated overnight at 37 °C, and the anti-QS effect was observed as an opaque inhibition zone around the paper disk, indicating the suppression of QS and pigment production.

### 2.6. Estimation of Violacein Pigment

To quantify violacein production, *C. violaceum* was exposed to varying BGNCs concentrations in 5 mL LB media and incubated at 30 °C for 24 h. The extraction of violacein pigment was followed by adding 10% sodium dodecyl sulfate for cell lysis and incubating for 10 min, and water-saturated n-butanol was added. The mixture was vortexed and centrifuged at 13,000 rpm for 15 min. The absorbance of the violacein-containing organic layer was measured at A585 nm.

### 2.7. Bacterial Survival Assay

To analyze the effect of BGNCs on *C. violaceum* and *P. aeruginosa* growth, 200 μL of each bacterial culture was inoculated into a 96-well plate and treated with sub-MICs of BGNCs [18]. Absorbance at 600 nm was recorded over 24 h in an ELISA plate reader with an automatic shaker at 37 °C. The experiment was performed in triplicate with appropriate controls.

### 2.8. Anti-QS Assays

#### 2.8.1. Assay for Biofilm Inhibition

The impact of newly developed BGNCs on PA01 biofilm formation was assessed using the MTP method [18]. Overnight-grown cells with biofilms in the MTP were stained with crystal violet, and absorbance was measured at 545 nm using a microtiter plate reader with OD values normalized to A595 nm. The percentage of PA01 biofilm inhibition was calculated using: % inhibition = [(ControlOD − TreatedOD)/ControlOD] × 100.

#### 2.8.2. Fluorescent Microscopic Biofilm Inhibition Assay

The anti-biofilm potential of NCs was evaluated on sterile glass coverslips, following [18]. Coverslips with biofilms were stained with live cells SYTO-9 and observed under a fluorescent microscope using green fluorescence. Additionally, biofilm cells were also stained with crystal violet (CV) and methylene blue (MB) solutions for 5 min, followed by rinsing three times with distilled water, and observed under a light microscope at 100× magnification to assess biofilm inhibition.

#### 2.8.3. Anti-Virulence Factors Assays

The PA01 bacteria, adjusted to an optical density of 2.0 at 600 nm, were inoculated into conical flasks containing nutrient broth and sub-MIC doses of the test samples. These flasks were incubated on a shaker at 37 °C for 24 h. After incubation, the cultures were centrifuged at 12,000 rpm for 15 min, and the supernatant was collected and filtered using Whatman filter paper. The filtered supernatant was subsequently used in anti-virulence experiments.

#### 2.8.4. Pyocyanin Quantification

Estimation of pyocyanin production was performed according to [18]. The culture supernatant was mixed with chloroform and vortexed. The upper layer was separated, acidified with 1 mL of 0.2 N HCl, and absorbance was measured at A520 nm.

#### 2.8.5. Pyochelin Estimation

Pyochelin was estimated by following [18] methodology. In brief, supernatant was mixed with 0.5 N HCL followed by nitrite molybdate and incubated at room temperature (RT) for 5 min. Further addition of NaOH and H_2_O and measured OD at A510 nm.

#### 2.8.6. Siderophore and Alginate Estimation

Quantification of Siderophore was done using 2% FeCl_3_, with absorbance measured at A445 nm. For the alginate, a colorimetric assay was performed. An equal volume of isopropanol and supernatant was mixed, incubated for 24 h, and then centrifuged. The precipitate was mixed with 10 mM boric acid dissolved in sulfuric acid and 0.2% carbazole reagent, incubated for 15 min at room temperature, and the OD was measured at A500 nm [19].

#### 2.8.7. Exopolysaccharide (EPS) Estimation

EPS quantification was done using heating the culture supernatant with 50% H_2_SO_4_. Subsequently, periodic reagents (1 mL), sodium arsenite (2 mL), and trichlorobutyric acid (2 mL) were added and further heated in a water bath. After cooling, butanol was added to separate the organic layer. Absorbance was measured at A548 nm [20].

#### 2.8.8. Protease Estimation

Protease enzyme production was quantified using the azocasein assay. A mixture of 2% azocasein in 50 mM Tris-HCl buffer (pH 7.8) with 2 mM CaCl_2_ and bacterial supernatant was incubated at 37 °C for 60 min. The reaction was stopped by adding 1.2 mL of 10% trichloroacetic acid and centrifugation at 8000 rpm for 10 min. Absorbance was measured at A440 nm. Additionally, protease activity was assessed using a modified media plate method as reported by [18]. Treated and untreated supernatants were applied to wells on the plate and incubated at 37 °C for 24 and 48 h, respectively. Alkaline protease production was indicated by a clear zone around the wells, with inhibition determined by comparison to the control [18,21].

#### 2.8.9. Rhamnolipid Estimation

To quantify rhamnolipid production by PA01, a modified orcinol assay [17]. The acidified supernatant (pH 2 with H_2_SO_4_) was mixed with methanol (1:2), vortexed, and the organic layer was dissolved in methanol. Next, 1% orcinol reagent (15% H_2_SO_4_) was added, and incubated at 80 °C for 30 min. Rhamnolipid production was quantified by measuring the optical density at A421 nm. In addition, rhamnolipid production on agar plates was assessed using a modified medium with sub-MIC samples at 37 °C for 24 h [18]. A yellowish-green zone around PA01 indicated rhamnolipid production interacting with CTAB.

#### 2.8.10. Swarming and Swimming Assay

To assess the ability of tested agents to inhibit swarming and swimming motility of *P. aeruginosa* PA01, overnight cultures were point-inoculated at the center of LB agar plates supplemented with 0.5% agar for swarming and 0.3% agar for swimming. Various sub-inhibitory concentrations of test samples were applied. Plates were then incubated at 37 °C for 48 h, and the diameters of swarming and swimming zones were measured [22].

#### 2.8.11. Assessment of BGNCs Effect on the Expression of Some Virulence Genes in *P. aeruginosa* (PA01) Using qRT-PCR

The ability of Nanocrystals to downregulate the expression of QS-controlled genes, namely *lasI*, *lasR*, *rhlI*, *rhlR*, *rhlA*, *rhlB*, *lasA*, and *lasB* in the standard strain *P. aeruginosa* Pa01 was assessed by qRT-PCR. The total bacterial RNA isolation was purified using TRIzol Reagent (QIAGEN) according to the manufacturer’s instructions. DNA contamination was eliminated through DNase treatment (Sigma). The RNA was quantified using agarose gel electrophoresis and the RNA 6000 Nanodrop analyzer techniques. The cDNA was produced using the Reverse Transcription System from Promega, along with random hexamers. The cDNA was amplified using the 7900HT real-time system (Applied Biosystem, Foster City, CA, USA) and SYBR Green (Thermo Fisher, USA) qPCR Master Mix. Primer sets for the experiment were designed with Primer 3 (version 0.4.0) [18,22]. The relative expression of the target genes was normalized to the housekeeping gene proC using the 2^−ΔΔCt^ method [23]. This experiment was carried out in triplicate.

#### 2.8.12. Statistical Analysis

Results are presented as the mean ± standard deviation from three independent experiments. Statistical significance was determined using a two-sample *t*-test to compare treated and untreated control groups. The data were recorded in triplicate. Graphs were created using GraphPad Prism software (version 8.0.1; GraphPad Software, Inc., San Diego, CA, USA).

## 3. Results

### 3.1. Characterization of NPs

**Figure 1:**
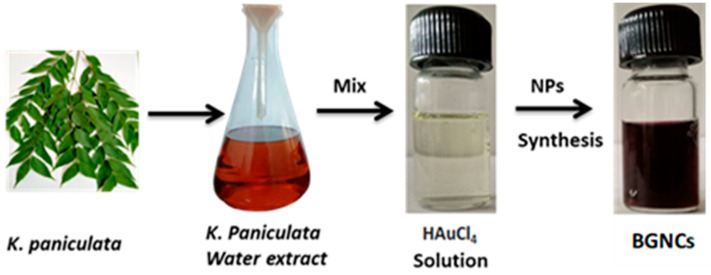
Fabrication of Biogenic nanocrystals (BGNCs). Representative image showing the results of the redox reaction after 30 min of storage, recorded both in the absence and presence of KP-Ext.

**Figure 2:**
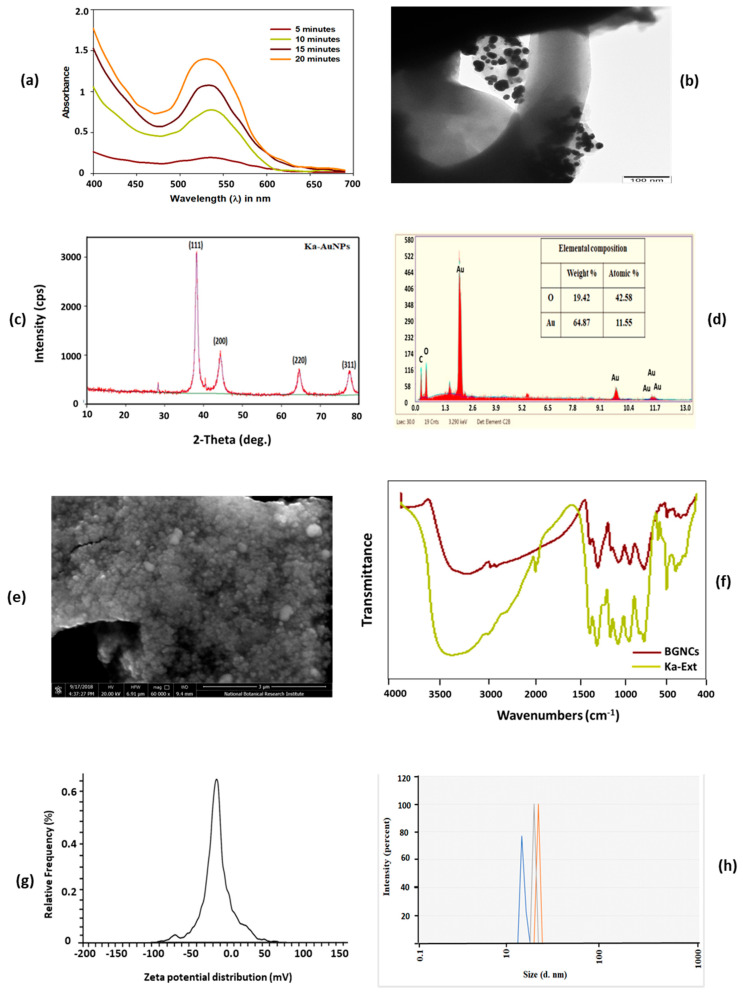
(**a**) Illustrating BGNCs UV–visible spectral analysis and their characteristic absorption profile Characterization profile of BGNCs. (**b**) TEM micrograph of BGNCs depicting the morphology and structural characteristics. (**c**) X-ray diffraction (XRD) pattern of BGNCs. (**d**) EDX spectrum of BGNCs. (**e**) SEM micrograph of BGNCs depicting the morphology. (**f**) Fourier-transform infrared (FTIR) spectra of KP-Ext and BGNCs. (**g**) Zeta potential of BGNCs showing overall charge. (**h**) Zeta size distributional of BGNCs showing average hydrodynamic diameter of particles. Different color peaks represent average intensity of different size NPs particles distributions.

Green-synthesized metallic nanoparticles are reduced and capped by secondary metabolites of plants; those have several enzymatic activities that can hamper nucleic acid synthesis, generate reactive oxygen species (ROS) in microorganisms [24]. Some unique properties of metallic nanoparticles, like biocompatible, large surface area, and stability that make them compatible with various clinical applications, such as cancer therapy, drug delivery, and eradication of microbial infections. Biogenic gold nanocrystals were synthesized using aqueous leaf extract of KP. BGNCs formation was visibly noticed by a change in color from light to ruby red after the addition of KP extract to the HAuCl_4_ solution, Figure 1. Further, BGNCs synthesis was authenticated via a UV-visible spectrophotometer by scanning spectra in the range of 380–700 nm. Figure 2a demonstrates a characteristic peak around 530–540 nm due to the excitation of surface plasmon vibration is indicating formation of BGNCs. This happened due to metal reduction by secondary metabolites present in the leaf extract. The absorption peaks at a specific wavelength were proportionally increasing with time.

BGNCs morphology and size distribution were determined by TEM. A quasi-spherical shape of BGNCs was observed with an average size of 10–30 nm Figure 2b. SEM analysis shows that the BGNCs were spherical and polydisperse with a narrow size distribution and an average size of 10–50 nm Figure 2e. EDX results exhibited that the predominant element is gold in ionic form. Carbon and oxygen peaks, along with gold, suggested that the organic moieties on the surface of nanoparticles may result from the extract Figure 2d.

The size distribution of BGNCs was analyzed using the DLS Zeta Sizer. As shown in Figure 2h, the average size distribution was 12 ± 1.3 nm with a PDI of 0.363. The zeta potential of the BGNCs was found to be negative −21 mV (Figure 2g). This negative charge contributes to the colloidal stability of BGNCs in a biological environment, preventing their accumulation.

The XRD analysis was incorporated to check the crystalline nature of BGNCs, Figure 2c. The investigation confirmed a face-centered cubic crystalline structure of gold, with peak positions at 2θ values of 38.3°, 44.4°, 64.5°, and 77.1°, corresponding to the lattice planes (111), (200), (220), and (311). FTIR analysis was conducted to determine the ionic interactions between KP leaf extract and the nanocrystals. The FTIR study revealed specific vibration band characteristics in the extract supernatant. Similar FTIR spectra were observed for BGNCs, indicating interactions between the phytochemicals and the developed nanocrystals Figure 2f. The FTIR spectra of BGNCs showed distinctive vibration bands at 3196.3, 2909.7, 2841.9, 1697.3, 1604.1, 1441.6, 1353.3, 1213.7, 1039.7, and 761.4 cm^−1^. The bands at 3196.3, 2909.7, and 2841.9 cm^−1^ represent the stretching vibrations of N-H, C-H, and O-H groups, while the bands in the range of 2000–1500 cm^−1^ indicate C=O, C=N, and C=C stretching vibrations. Bands in the range of 1400–1000 cm^−1^ correspond to C-F, C-H, and C-O stretching in polysaccharide groups, Figure 2f.

### 3.2. QS Inhibition Potential of BGNCs

**Figure 3:**
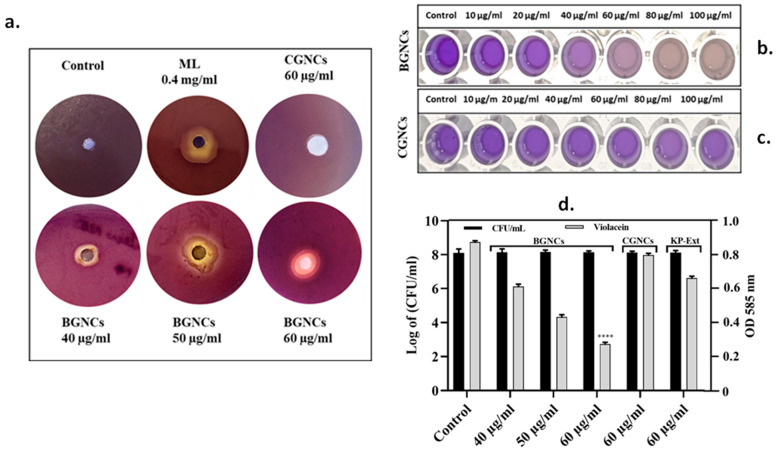
(**a**) Bioindicator bacterial strains were employed to evaluate the anti-QS potential of BGNCs. The assay was performed using *Chromobacterium violaceum* 12472 treated with BGNCs (60 μg/mL), their chemical analogs CGNCs (60 μg/mL), and the leaf extract of *K. paniculata* (KP-Ext, 60 μg/mL). Menthol (ML, 0.4 mg/mL) served as the positive control, while distilled water (DW) was used as the untreated control. (**b**,**c**) The effect of varying concentrations of BGNCs and CGNCs on violacein production by *C. violaceum* 12472 was assessed under broth culture conditions. (**d**) The bacterial growth phase and corresponding inhibition of violacein synthesis following treatment with KP-Ext, BGNCs, and CGNCs were quantified spectrophotometrically. Data represent the mean ± standard deviation from three independent experiments. **** *p* < 0.0001 compared to the control.

The quorum-sensing inhibition potential of BGNCs was assessed using the bio-indicator strain *Chromobacterium violaceum* 12472 through a disk diffusion assay (Figure 3). *C. violaceum* produces the violet pigment violacein, regulated by AHL-based quorum sensing (QS). Therefore, inhibiting pigment production without impacting bacterial growth indicates potential QS inhibition. The results demonstrated a concentration-dependent inhibitory zone for violacein biosynthesis in *C. violaceum* at BGNCs levels between 40 and 60 µg/mL (Figure 3a,b). The best results were exhibited at 60 µg/mL for BGNCs as compared to CGNCs (60 µg/mL) and the control. A positive control: Menthol (0.4 mg/mL) also showed a similar inhibitory zone for violacein biosynthesis. A similar opaque zone of QS inhibition was examined with the KP leaf extract treatment at a higher concentration, while there was no inhibition in CGNCs (Figure 3a,c). In addition, concentration-dependent suppression of violacein pigment was also quantified by a UV-vis spectrophotometer. The most significant results were obtained at a 60 µg/mL concentration of BGNCs, compared to CGNCs at 60 µg/mL, without affecting bacterial growth (Figure 3b–d). This could be possible due to interference with QS of *C. violaceum*.

### 3.3. Biofilm Inhibition Potential of BGNCs

**Figure 4:**
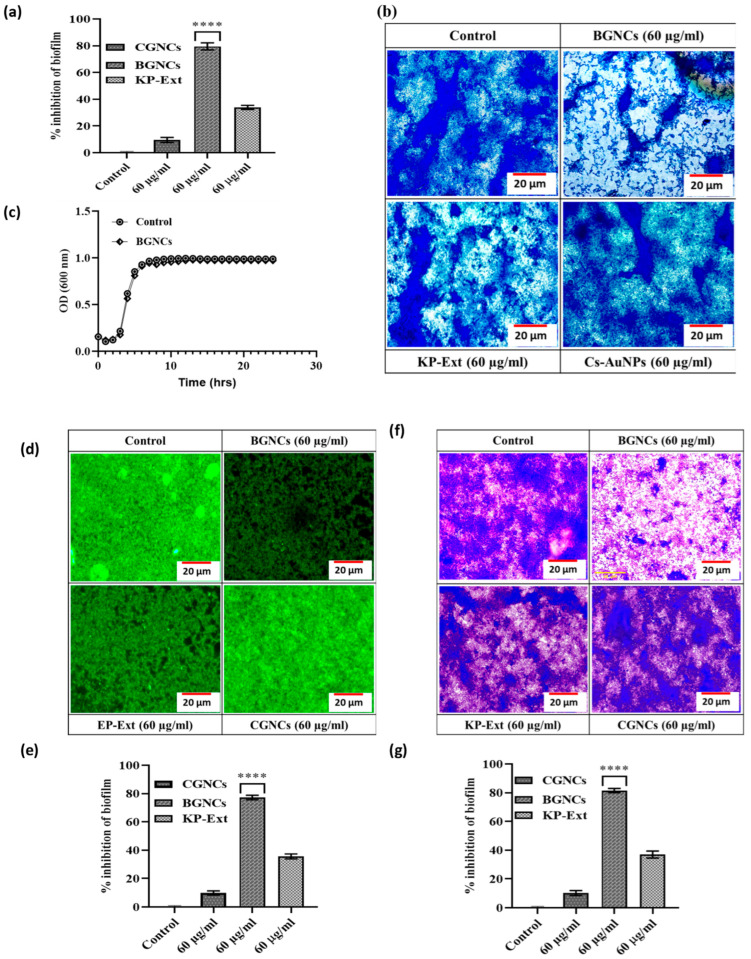
Anti-biofilm activity of BGNCs against *P. aeruginosa* PA01. (**a**,**e**,**g**) Biofilm inhibition by KP-Ext, BGNCs, and CGNCs (60 μg/mL) on *P. aeruginosa* PA01 was quantified spectrophotometrically by CV staining assay after 24 h. Data are the mean ± SD (*n* = 3), **** *p* < 0.0001 vs. control. (**c**) Effect of BGNCs on the cells of *P. aeruginosa* PA01, measured using growth kinetics. The graph indicates the mean and standard deviation of three independent replicates. (**b**,**d**,**f**) Microscopy images of biofilms on cover glass treated with (**b**) MB, (**d**) SYTO-9, and (**f**) CV staining. Scale bar: 20 μM.

QS-signaling also regulates bacterial biofilm development. Hence, the efficacy of BGNCs on biofilm development in *P. aeruginosa* was quantitatively examined by a typical crystal violet test in 96-well polystyrene plates. The most promising results were obtained at sub-MIC levels of BGNCs compared to CGNCs (Figure 4). Treatment with the highest concentration (60 µg/mL) of BGNCs resulted in a greater than 80% reduction in biofilm development in PA01 after 24 h of incubation (Figure 4a,b). Bacterial adhesion to any surface was apparently necessary for the formation of biofilms. To assess the inhibitory effects of BGNCs on bacterial adhesion, the BGNCs-treated glass coverslips were incubated with the bacterial culture for 24 h at 37 °C. The fluorescent microscopic images of *P. aeruginosa* PA01 biofilms, both treated and untreated with BGNCs, are exhibited in Figure 4d. These images reveal a noticeable reduction in biofilm development at the BGNCs concentration of 60 µg/mL (Figure 4e). Figure 4b,f shows a thick biofilm on the untreated control’s glass surface. Bacteria were found to be strongly colonized on the glass surface, with a heavy accumulation of cells in biofilms. Treatment with BGNCs resulted in the reduction in biofilm on glass coverslip. Additionally, it was noted that the cells were dispersed and that only a small number of them were capable of colonization. Figure 4b,f shows the light microscopic micrographs of *P. aeruginosa* biofilms under treated and untreated control conditions. In the untreated control, bacteria were heavily colonized on the surface, producing a thick clump of cells. The capacity of PA01 to generate biofilms was considerably diminished when the cultures were treated with BGNCs. The microscopic images show that the bacterial colonization and adhesion to the glass surface were significantly reduced Figure 4b,d,f. The findings show that the sub-MIC of BGNCs was a typical concentration for anti-biofilm activity. Hence, Sub-MIC were tested on the growth kinetics of PA01, resulting in no substantial shift in cell population after 32 h of incubation as compared to the untreated control (Figure 4c). Finally, it was discovered that BGNCs were more effective than CGNCs in suppressing biofilm formation without impacting PA01 cell survival.

### 3.4. QS-Regulated Virulence Factor Inhibition Potential of BGNCs

**Figure 5:**
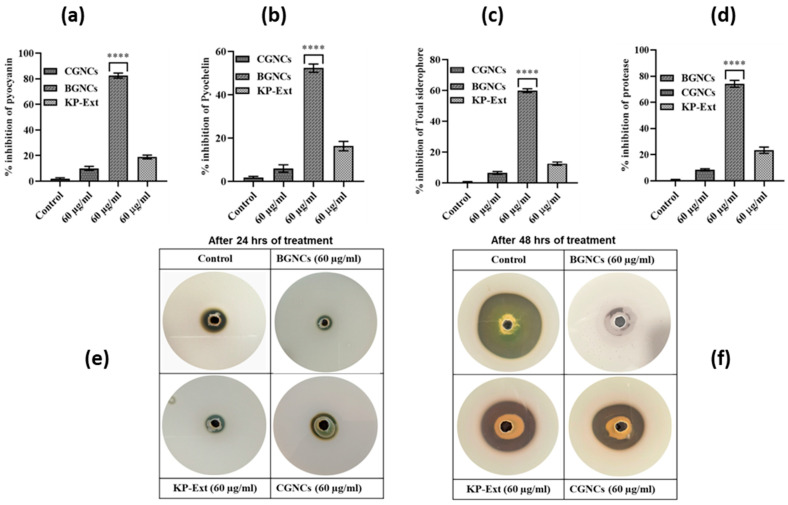
Effect of BGNCs on the production of quorum-sensing (QS)-regulated virulence factors in *Pseudomonas aeruginosa* PAO1. The levels of QS-controlled virulence factors—(**a**) pyocyanin, (**b**) pyochelin, (**c**) total siderophore, and (**d**) protease—were quantified from cell-free culture supernatants of PAO1 treated with 60 μg/mL of KP-Ext, BGNCs, and CGNCs for 24 h. The results are presented as the percentage inhibition of virulence factor production in vitro. (**e**,**f**) Visualization of protease inhibition on casein agar plates showing clear zones indicating proteolytic activity after (**e**) 24 h and (**f**) 48 h incubation with the respective treatments. Data are expressed as the mean ± standard deviation (SD) from three independent experiments. **** *p* < 0.0001 compared to the control.

In *P. aeruginosa*, QS controls many virulence factors that increase pathogenicity by impairing the cellular functions of the host immune system. The effect of BGNCs on various QS-regulated virulence factors in PA01 was estimated spectrophotometrically. BGNCs showed a concentration-dependent reduction in virulence factors compared to the CGNCs and control. The prominent results were obtained at a con. 60 µg/mL of BGNCs: pyocyanin (73%), pyochelin (52%), and total siderophore (59%) (Figure 5a–c). BGNCs were notably more effective in down-regulating the virulence factors as compared to their chemical counterparts and plant extract. At a concentration of 60 µg/mL, BGNCs-treated PA01 cells exhibited significantly reduced azocasein-degrading protease activity (74%) compared to the untreated control and CGNCs Figure 5d. Enzyme Protease production was assessed using an agar plate assay in a time-dependent manner, which demonstrated a statistically significant reduction in enzymatic activity in the BGNCs-treated group compared to the CGNCs and control Figure 5e,f.

**Figure 6:**
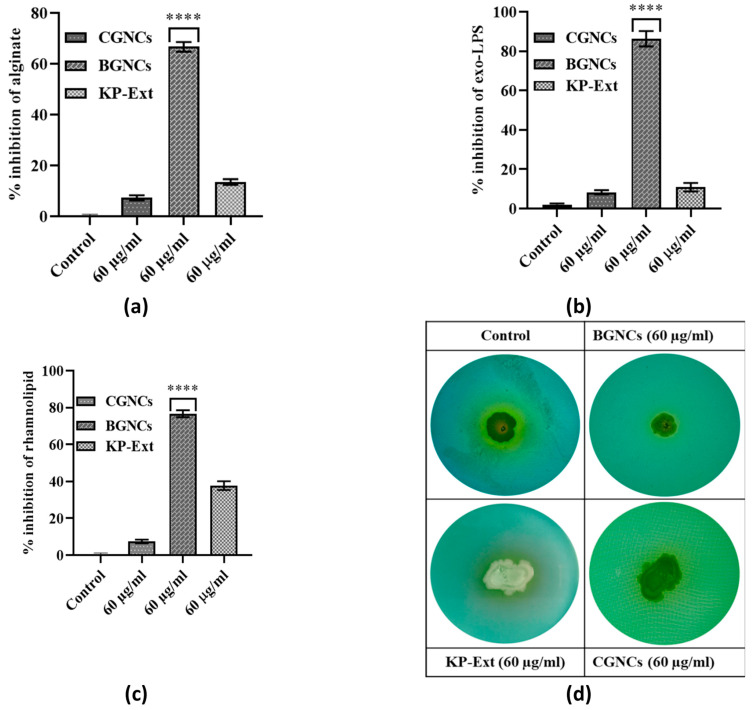
Effect of BGNCs on the production of quorum sensing (QS)-regulated virulence factors in *Pseudomonas aeruginosa* PAO1. The levels of QS-controlled virulence factors—(**a**) alginate, (**b**) exopolysaccharides (exo-LPS), and (**c**) rhamnolipids—were quantified from cell-free supernatants of PAO1 cultures treated with 60 μg/mL of KP-Ext, BGNCs, or CGNCs for 24 h. The data represent the percentage inhibition of virulence factor production in vitro. (**d**) Evaluation of rhamnolipid synthesis by the Petri plate assay, where the appearance of a yellow zone around bacterial colonies indicates rhamnolipid production. The graph displays the mean ± standard deviation from three independent experiments. **** *p* < 0.0001 compared to the control.

Alginate, an exopolysaccharide regulated by quorum sensing (QS), is an integrant part of the biofilm matrix in PA01. BGNCs significantly reduced alginate production by 66% at a 60 µg/mL concentration Figure 6a. In addition, Exo-LPS production decreased by 82% at a similar concentration Figure 6b. Another virulence factor, rhamnolipid, is known to play a crucial role in QS-regulated swarming motility and biofilm dispersion at the infection site. Rhamnolipid production was decreased by over 76% at an identical concentration of BGNCs Figure 6c. Furthermore, inhibition of rhamnolipid production was also confirmed using a treated culture supernatant of PA01 in an agar plate assay (Figure 6d). The agar plate assay confirmed the reduction in rhamnolipid production, supporting the data that BGNCs effectively diminish virulence factor production in *P. aeruginosa* PA01. Therefore, results indicate that BGNCs significantly inhibit the production of virulence factors.

**Figure 7:**
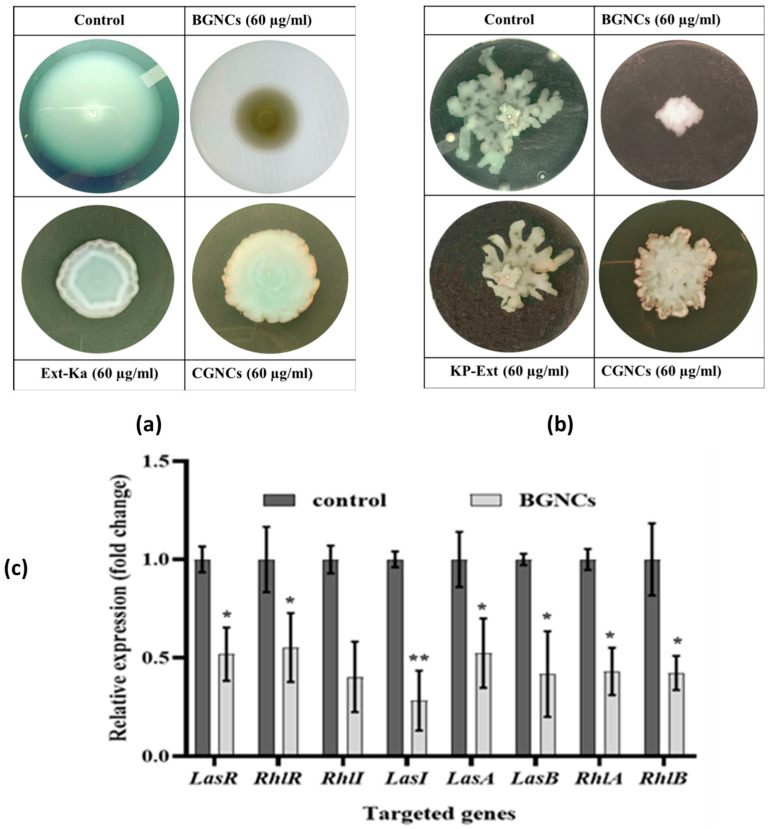
BGNCs’ effect on the swarming and swimming motilities of PA01. Effect of indicated test samples and their concentrations on (**a**) swimming and (**b**) swarming motilities of *P. aeruginosa* PA01 on solid agar medium. (**c**) BGNCs’ effect on the QS-regulated gene expression in *P. aeruginosa* PA01. The graph indicates the mean and standard deviation of three independent replicates. * *p* < 0.05 vs. control. ** *p* < 0.01 vs. control.

Bacterial motility significantly contributes to the spread of bacterial infections. To assess the migration ability of PA01 on an agar plate with and without test samples, a swimming and swarming assay was conducted. BGNCs showed a notable impact on bacterial motility compared to CGNCs and the untreated control, respectively, Figure 7a,b. The results indicated that sub-MICs of BGNCs inhibit QS-regulated motility by suppressing rhamnolipid genes, effectively controlling the flagellum-driven motility of PA01. Quantitative data are represented in Table 1.

### 3.5. Down-Regulation of QS-Associated Virulence Genes by BGNCs

The anti-QS effect of BGNCs on QS-regulated virulence factors and biofilm gene expression in PA01 was evaluated using qRT-PCR. Treatment with BGNCs at a sub-MIC of 60 µg/mL significantly down-regulated the gene expression in PA01 Figure 7c. The expression levels of QS-related genes such as lasA, lasB, lasI, rhlA, rhlB, rhlR, rhlI, and pqsC were measured in BGNCs-treated PA01 cells. All genes of interest showed a significant reduction in expression, ranging from 0.07946 to 2.26577-fold compared to the untreated control Figure 7c. The housekeeping gene proC was not significantly affected by BGNCs treatment.

## 4. Discussion

*Pseudomonas aeruginosa* biofilm represents a significant barrier in clinical treatments due to its remarkable resistance to various antimicrobial agents. The biofilm matrix acts as a protective barrier against antibiotics and host immune responses. Microorganisms capable of biofilm formation typically share common traits, including the production of extracellular polymeric substances (EPS) and strong intercellular adhesion. Among *P. aeruginosa*’s virulence determinants, pyocyanin a redox-active pigment plays a crucial role in pathogenicity and biofilm development. Its production is regulated by quorum sensing (QS), a bacterial communication system that coordinates the expression of virulence genes in response to population density [25,26,27]. Given the increasing challenge of antimicrobial resistance, medicinal plants and their phytochemicals offer promising therapeutic alternatives due to their diverse bioactive compounds and biocompatibility [28,29,30,31]. Complementing this, green nanotechnology has emerged as an innovative and sustainable approach in biomedical applications [32]. Nanoparticles provide new avenues for combating multidrug resistance and biofilm-associated infections through their unique physicochemical properties. This study investigates the potential of biogenic gold nanocrystals (BGNCs) in modulating QS-regulated virulence factors and biofilm formation in *P. aeruginosa* PAO1. Gold nanoparticles are highly valued in nanomedicine due to their excellent stability, biocompatibility, and ease of synthesis [33]. They exhibit antibacterial, antiviral, and antiparasitic activity without causing cytotoxic effects on human cells and have been applied in drug delivery, bioimaging, diagnostics, and photodynamic cancer therapy [34,35,36,37,38]. In this study, *Koelreuteria paniculata* leaf extract was employed for the green synthesis of BGNCs. The formation of nanocrystals involves the reduction and stabilization of gold ions by plant-derived secondary metabolites such as phenolics, alkaloids, polysaccharides, terpenoids, and polyphenols. These biomolecules act as reducing and capping agents, ensuring stable nanostructure formation with enhanced biological activity.

The QS inhibitory efficacy of biogenic gold nanocrystals was checked in vitro by using a bio-indicator strain *C. violaceum* 12472. It stated that *C. violaceum* regulates violacein production via AHL-mediated QS [17]. The results demonstrate that the inhibition of violacein production by BGNCs occurs in a concentration-dependent manner (Figure 3). Approximately 80% inhibition of violacein production was observed with 60 µg/mL BGNCs, consistent with previous studies on nanomaterials’ effects against QS and violacein production [18]. Chemically synthesized counterparts of gold nanocrystals showed very low or no inhibition at the same concentration (Figure 3a). *P. aeruginosa*, a common lung infection pathogen, causes significant morbidity and mortality in cystic fibrosis patients [39]. To prevail over human health, it uses bacterial crosstalk, or QS, which serves as a director for the creation of virulence factors (VFs). BGNCs significantly inhibit virulence factor production and biofilm formation, including the synthesis of signaling molecules and QS-regulated genes in PA01, by reducing the principal las-rhl pathways. The lasI and rhlI genes are integral to the lasR and rhlR systems, which constitute the AHL network and govern the production of various virulence factors, including LasA protease and LasB elastase, and the processes of motility and biofilm formation [40]. The rhl system also controls the production of rhamnolipid, which possesses both hemolytic and bio-surfactant properties [41] and plays a crucial role in acute PA01 infections [42]. Supporting the anti-QS theory, BGNCs significantly reduced the synthesis of VFs such as pyocyanin, pyochelin, alginate, and rhamnolipid (Figure 5a,b and Figure 6a,c,d). The observed suppression of lasR expression, which regulates AHL production, aligns with the decreased production of iron-chelating siderophores and proteases (Figure 5c–f). Moreover, the study revealed that BGNCs effectively diminished the production of rhamnolipids, as shown in Figure 6c,d, suggesting a potential impact on the regulation of the rhl operon. This highlights the potential of BGNCs to mitigate PA01 virulence factors and biofilm formation. Biofilms serve as protective shields for bacterial cells, rendering them over 1000 times more resistant to antibiotics compared to their planktonic counterparts. Notably, biofilms are recognized as pivotal contributors to chronic infections [43,44]. Consequently, the deactivation of quorum sensing corresponds to a reduction in biofilm formation in PA01. The results clearly demonstrate the significant inhibitory effects of BGNCs on biofilm formation and microcolony development, correlated with the concentration of BGNCs (Figure 4). As earlier reports described similar results of QS inhibitors [45,46,47]. Furthermore, the decline in alginate and rhamnolipids, integral components of the extracellular matrix of PA01 biofilms, exhibited a notable correlation with alterations in the biomass of biofilm (Figure 6a,c,d). Intriguingly, BGNCs treatments induced a noteworthy impact on the expression of rhamnolipid genes (rhlA and rhlB) in PA01 (as shown in Figure 7c).

Additionally, the assessment of biofilm quantity also confirmed the effective suppression of static PA01 biofilm biomass by BGNCs (Figure 4). Similarly, the motility assay demonstrated a substantial reduction in both swimming and swarming motility of PA01 due to the treatment of BGNCs (Figure 7a,b). Notably, flagella-mediated movement plays a pivotal role in the initial stages of biofilm formation by aiding in attachment and surface colonization [48]. Consequently, the observed suppression of swimming and swarming motility is closely linked to the inhibition of biofilm formation, as reported in prior studies (quantitative data represented in Table 1) [47,49]. The impact of BGNCs results in reduced biofilm thickness, thereby increasing the population of planktonic cells in the medium without the protective EPS-matrix, rendering them more susceptible to antibiotics. Previous research suggests the potential of anti-QS compounds and plant extracts in enhancing antibiotic efficacy against mature PA01 biofilms.

RT-qPCR analysis revealed substantial downregulation of key QS genes, including lasI, lasR, rhlI, rhlR, and rhlA in BGNCs-treated *P. aeruginosa* PAO1 compared with untreated controls. Normally, as bacterial density increases, the LasI-LasR complex functions as a transcriptional activator to regulate virulence-associated genes. BGNCs exposure disrupted this regulatory loop, resulting in the suppression of QS-signaling components and their downstream effectors. The quorum-sensing genes lasI encoding the LasI synthase that produces the signal molecule 3OC12-HSL and lasR encoding the LasR receptor and transcriptional activator that binds 3OC12-HSL were significantly downregulated following BGNCs treatment. This suggests that both the production of the signaling molecule and receptor-mediated activation were effectively blocked by BGNCs. Overall, this study establishes that biogenically synthesized gold nanoparticles (BGNCs) derived from *K. paniculata* leaf extract effectively inhibit quorum sensing and biofilm formation in *P. aeruginosa* PAO1. By downregulating las and rhl QS systems and their associated virulence genes, BGNCs exhibit potent anti-QS, anti-virulence, and antibiofilm properties.

## 5. Conclusions

In our detailed investigation of BGNCs’ impact on quorum sensing (QS), we assessed QS gene expression and the translation of QS-signaling molecules. Our findings revealed the downregulation of conventional QS pathways, particularly Las-Rhl in PA01 (see Figure 7c). As cell density reaches a critical point, the LasI-LasR complex acts as a transcriptional activator, controlling key genes such as LasA, LasB, LasI, LasR, RhlR, and RhlA. RT-qPCR analysis revealed a significant decrease in gene expression compared to the untreated control. This suggests that BGNCs may have a broad inhibitory effect on PA01 QS signaling and its components.

## Figures and Tables

**Table 1:** BGNCs’ effect on the swarming and swimming motilities of PA01. ** *p* < 0.01 vs. control. *** *p* < 0.001 vs. control.

Motility Assay			
S. No.	Test Sample	Swarming Motility	Swimming Motility
		Diameter (mm) of Bacterial Colony at Different Concentrations of Test Samples	Diameter (mm) of Bacterial Colony at Different Concentrations of Test Samples
1	Control	65 ± 5.29	64 ± 4.47
2	KP-Ext 60 µg	40 ± 4.85	41 ± 3.94
3	BGNCs 60 µg	23 ± 3.01 ***	30 ± 3.15 **
4	CGNCs 60 µg	56 ± 3.88	53 ± 3.75

## Data Availability

No data were used for the research described in the article.
